# Idiopathic granulomatous hypophysitis presenting with galactorrhea, headache, and nausea in a woman: a case report and review of the literature

**DOI:** 10.1186/s13256-019-2276-4

**Published:** 2019-11-16

**Authors:** Guive Sharifi, Mohammad Reza Mohajeri-Tehrani, Behrouz Navabakhsh, Bagher Larijani, Touraj Valeh

**Affiliations:** 1 Department of Neurosurgery, Loghman Hakim Hospital, Shahid Beheshti University of Medical Sciences, Tehran, Iran; 2 Endocrinology & Metabolism Research Center, Endocrinology & Metabolism Clinical Sciences Institute, Tehran University of Medical Sciences, Dr. Shariati Hospital, Jalal Al Ahmad Highway, Tehran, 1411713137 Iran

**Keywords:** Idiopathic granulomatous hypophysitis, Autoimmune hypophysitis, Pituitary gland, Galactorrhea, Transsphenoidal surgery

## Abstract

**Background:**

Inflammation of the pituitary gland can occur in a variety of primary or secondary disorders. Idiopathic granulomatous hypophysitis is a rare inflammatory disease of the pituitary gland that can closely mimic a pituitary adenoma clinicoradiologically. Most authorities agree on minimally invasive transsphenoidal surgery as the mainstay in diagnosis and treatment of this disorder. There is still some controversy regarding pure medical management of idiopathic granulomatous hypophysitis in the literature.

**Case presentation:**

A 47-year-old Iranian woman of Azeri ethnicity with a history of benign breast cysts with a chief complaint of galactorrhea presented to our endocrinology clinic. Her past medical history was negative for any menstrual irregularities, hirsutism, visual complaints, diplopia, polyuria and polydipsia or seizures. She was taking 100 mcg of levothyroxine daily. Her familial history and physical examination were unremarkable. Her initial laboratory work-up revealed hyperprolactinemia (82.4 ng/mL) with otherwise normal pituitary axes. Brain magnetic resonance imaging showed a pituitary macroadenoma for which she was treated with 0.5 mg of cabergoline weekly. Although her serum prolactin level dropped to 1.7 ng/mL and her galactorrhea was resolved, she continued to complain of headaches and nausea. Repeated imaging showed no decrease in size of the macroadenoma. Therefore, she underwent transsphenoidal surgery of the macroadenoma which was reported as chronic granulomatous hypophysitis by expert pathologists. Tuberculosis, sarcoidosis, Wegener’s granulomatosis, Langerhans cell histiocytosis, and syphilis were ruled out by appropriate tests and she was diagnosed as having idiopathic granulomatous hypophysitis. Fortunately, her condition was not complicated by hypopituitarism and she was symptom free 9 months after transsphenoidal surgery.

**Conclusions:**

Idiopathic granulomatous hypophysitis, a rare inflammatory disease of the pituitary gland, is a diagnosis of exclusion for which both medical and surgical management are reported in the literature. We present a case of idiopathic granulomatous hypophysitis who was symptom free with no complications of hypopituitarism following its transsphenoidal resection after 9 months of follow-up.

## Background

Inflammation of the pituitary gland can occur in a variety of primary or secondary disorders. Idiopathic granulomatous hypophysitis (IGH) is a rare inflammatory disease of the pituitary gland that can closely mimic a pituitary adenoma clinicoradiologically. Most authorities agree on minimally invasive transsphenoidal surgery (TSS) as the mainstay in diagnosis and treatment of this disorder. There is still some controversy regarding pure medical management of IGH in the literature [1, 2].

The scarcity of reports on IGH in the literature resulted in a dilemma in its treatment. Therefore, we describe a case of IGH presenting with galactorrhea, headaches, and nausea and discuss it briefly with a review of the published literature on similar cases.

## Case presentation

A 47-year-old Iranian woman of Azeri ethnicity with a history of benign breast cysts and hypothyroidism was referred to our endocrinology clinic for further evaluation of her new onset galactorrhea. She also complained of occasional headaches and nausea. She did not report any menstrual irregularities, hirsutism, visual complaints, diplopia, polyuria and polydipsia or seizures. She was a housewife with no history of occupational exposure and her familial history was unremarkable. She had never smoked tobacco or consumed alcohol. At admission, her blood pressure was 110/75 mmHg, pulse rate was 85 beats per minute, and temperature was 36.8 °C orally. A physical examination did not reveal any visual field defects and on fundoscopy both eyes were normal with sharp optic disc margins. Her visual acuity was 10/10 bilaterally. Other neurological examinations including cranial nerves were unremarkable. Her initial laboratory work-up showed a moderately elevated serum prolactin level (Table 1). A hormonal assay did not show any impairments in other pituitary axes and her renal function was normal (Table 1). She was not taking any medications other than levothyroxine 100 mcg daily at that time.

**Table 1:** Laboratory data

Laboratory data	Value	Normal range
White blood cell	9400/mm^3^	4500–11,000/mm^3^
Hemoglobin	14.6 g/dL	11.8–15.8 g/dL
Mean corpuscular volume	97.1 fL	81–100 fL
Platelets	253,000/mm^3^	150,000–450,000/mm^3^
Aspartate aminotransferase	21 U/L	Up to 32 U/L
Alanine aminotransferase	19 U/L	Up to 35 U/L
Alkaline phosphatase	165 U/L	98–279 U/L
Creatinine	0.9 mg/dL	0.5–1.2 mg/dL
Urine analysis	Normal	
Prolactin	82.4 ng/mL	Up to 29.5 ng/mL
Thyroid-stimulating hormone	5.91 μIU/mL	0.4–6.0 μIU/mL
Thyroxine (T4)	6.45 μg/dL	4.5–12.6 μg/dL
Triiodothyronine (T3)	0.86 ng/mL	0.6–2.2 ng/mL
Adrenocorticotrophic hormone	22 pg/mL	9–52 pg/mL
Insulin-like growth factor 1	149 ng/mL	94–252 ng/mL
Cortisol 8 a.m.	11.0 μg/dL	6.2–20 μg/dL
Luteinizing hormone	1.82 mIU/mL	0.5–10.5 mIU/mL
Follicle-stimulating hormone	4.6 mIU/mL	3.0–12.0 mIU/mL
Lactate dehydrogenase	595 U/L	230–460 U/L
Angiotensin-converting enzyme	24.0 U/L	13.30–63.90 U/L
Ferritin	87.3 ng/mL	10–124 U/L

**Fig. 1:**
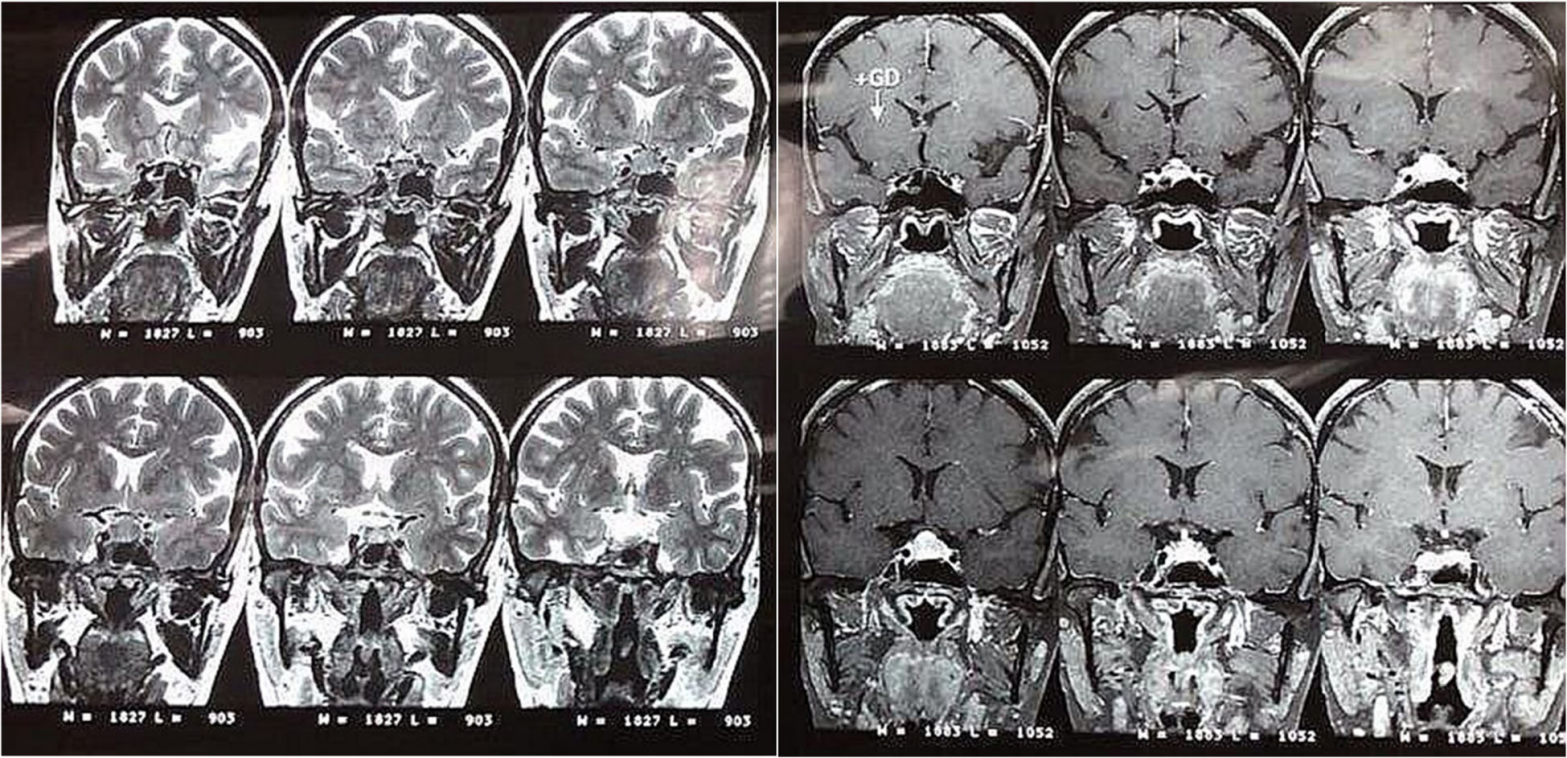
Pituitary magnetic resonance imaging without and with gadolinium enhancement showing an intermediate signal intensity mass of approximately 16 × 11 mm dimensions within the pituitary gland extending to suprasellar cistern causing mild mass effect on optic chiasma compatible with pituitary macroadenoma. Cavernous sinus appears normal and there is no evidence of invasion

She underwent a dynamic 1.5 T magnetic resonance imaging (MRI) of the brain and hypophysis with images obtained every 28 seconds after injecting contrast material. The MRI showed a pituitary macroadenoma measuring 16 × 11 mm extending to the suprasellar cistern causing mild mass effect on optic chiasm (Fig. 1). She was started on cabergoline 0.5 mg weekly and after 1 month her serum prolactin was lowered to 1.7 ng/mL, and her galactorrhea was resolved. Her thyroid-stimulating hormone (TSH), adrenocorticotrophic hormone (ACTH), and insulin-like growth factor 1 (IGF1) were rechecked and found to be normal again. Despite her laboratory data, our patient continued to complain of headaches and nausea. A perimetric examination did not show any significant visual field defects. A repeated MRI approximately 3 months after her initial one showed no decrease in size of the macroadenoma. She was referred to a neurosurgeon who deemed her persistent symptoms and macroadenoma reason enough for performing TSS. Prednisolone and levothyroxine were prescribed and the mass was removed and reported twice by expert pathologists as chronic granulomatous hypophysitis with negative acid-fast and periodic acid–Schiff (PAS) stains for tubercle bacilli and fungal elements. No eosinophils or features of Langerhans cell histiocytosis were reported.

Tuberculosis was ruled out in our patient by a negative tuberculin skin test, chest X-ray (CXR), and polymerase chain reaction of cerebrospinal fluid and hypophysis specimen. She did not complain of any pulmonary symptoms and a normal CXR and angiotensin-converting enzyme levels ruled out sarcoidosis as well. Serologic studies for Wegener’s granulomatosis and syphilis came back as negative. Therefore, our patient was diagnosed as having IGH. She was closely followed up postoperatively under the care of her neurosurgeon and endocrinologist with periodic office visits and laboratory tests of pituitary axes and a postoperative dynamic MRI of the sella turcica region. At 9 months of follow-up, serum levels of TSH, thyroxine (T4), gonadotropins, prolactin, cortisol, IGF1, and ACTH were normal. Moreover, her MRI did not reveal any abnormal growth or mass and she was free of headache. Fortunately, our patient’s condition was not complicated by hypopituitarism and she was symptom free 9 months after TSS.

## Discussion and conclusions

IGH, a rare inflammatory disease of the pituitary gland, is a diagnosis of exclusion for which both medical and surgical management are reported in the literature. We described a case of IGH presenting with galactorrhea, headaches, and nausea who did not respond to medical treatment and underwent transsphenoidal resection. She was symptom free with no complication of hypopituitarism after 9 months of follow-up.

Inflammation of the pituitary gland can occur as a primary phenomenon or secondary to various systemic diseases. The diagnosis of primary hypophysitis is made following exclusion of secondary causes and can be subdivided based on histologic findings into five different subtypes: lymphocytic, granulomatous, xanthomatous, xanthogranulomatous, and necrotizing [3]. IGH is second to lymphocytic hypophysitis in prevalence. Secondary causes of granulomatous hypophysitis include tuberculosis, sarcoidosis, syphilis, pituitary adenoma, Langerhans cell histiocytosis, Wegener’s granulomatosis, and Rathke’s cleft cyst rupture [1].

The majority of cases of IGH become clinically apparent with headaches, nausea, and visual and oculomotor disturbances due to mass effects from an enlarged pituitary gland, prompting the need for surgical resection of the gland. It follows, therefore, that a large number of cases of IGH may be subclinical or manifest in ways that are not attributable to mass effects. The most clinically significant manifestations are central diabetes insipidus (DI) and hypopituitarism. IGH may account for a portion of cases of hypopituitarism and central DI for which treatment is issued without knowing the cause [1]. The symptoms in our case were attributed to the enlargement of the pituitary gland, causing a modestly high level of serum prolactin secondary to stalk compression.

Different studies have reported different rates of endocrine abnormalities in primary hypophysitis. In a review of 22 cases by Park *et al.*, infundibuloneurohypophysitis was the most common subtype, either isolated or combined with anterior hypophysitis, with 18 out of the 22 reviewed cases (82%) suffering from DI [2]. Hunn *et al*. reviewed 82 cases among whom suppression of hypothalamus–pituitary–adrenal axis was the most common (73%) while DI rate was reported as 27% [1]. Increased prolactin levels were found in 23% and 52% of the cases reviewed in these studies respectively [1, 2]. In a review of 379 cases of primary lymphocytic hypophysitis, anterior pituitary hormones were deficient in 90% of cases while 35% developed DI. There is still some debate concerning whether lymphocytic and granulomatous hypophysitis are different clinical entities or temporarily distinct parts of the same disease process in the course of which lymphocyte-dominant inflammation occurs earlier and granulomatous changes appear later [4].

Granulomatous hypophysitis closely mimics a pituitary adenoma clinicoradiologically. Most of the time, a diagnosis is only reached after histopathological studies. Most cases described so far have therefore been diagnosed after surgery. It has been postulated, however, that if one suspects hypophysitis before surgery, improvement following steroid therapy may help in differentiating the two [5]. A thickened stalk on MRI has been cited as the most characteristic radiology finding in favor of hypophysitis [6].

Current literature suggests that suspected inflammatory lesions of the pituitary should be managed conservatively and surgery should be performed only in the presence of serious and progressive deficits of visual fields, visual acuity, or ocular movements that are not responsive to medical treatment [2]. There have been several reports of satisfactory response to steroid and immunosuppressive treatments [7]. Xu *et al.* posited that pure glucocorticoid treatment is probably less effective in granulomatous hypophysitis than in lymphocytic hypophysitis and recommended it only as an adjuvant to minimally invasive surgery [8]. Hunn *et al*. reviewed 82 cases of IGH [1]. Among 64 cases for whom data on treatment modalities were available, the majority were treated with excisional pituitary surgery alone (46.9%, *n* = 30/64), excisional surgery and corticosteroids (35.9%, *n* = 23/64), or pituitary biopsy and corticosteroids (14.1%, *n* = 9/64). The authors did not find statistically significant differences in needs for hormone replacement therapy or recurrence rates between different treatment modalities [1].

Normal pituitary tissue is usually preserved at the hands of experienced pituitary neurosurgeons and the need for long-term hormonal replacement is predicted by panhypopituitarism at presentation. If there is concern that complete removal of the mass may compromise an otherwise preserved pituitary function, it is reasonable to diagnose hypophysitis with transsphenoidal biopsy and fast frozen pathology, since the condition is amenable to corticosteroid therapy [3]. Galactorrhea, hyperprolactinemia, normal gonadal axis, and euthyroidism at presentation have all been associated with reduced requirement for long-term hormone replacement [1]. Stereotactic radiotherapy as reported by Selch *et al.* has been successfully employed in the treatment of recurrent lymphocytic hypophysitis [9] and it can be considered a potential noninvasive treatment of granulomatous hypophysitis especially in cases of disease recurrence or resistance to glucocorticoids.

## Data Availability

All of the data and materials will be available upon a reasonable request to the corresponding author.

## Abbreviations

ACTH: Adrenocorticotrophic hormone
CXR: Chest X-ray
DI: Diabetes insipidus
IGF1: Insulin-like growth factor 1
IGH: Idiopathic granulomatous hypophysitis
MRI: Magnetic resonance imaging
TSH: Thyroid-stimulating hormone
TSS: Transsphenoidal surgery
